# Evaluation of risk-based antigen and antibody surveillance strategies and their association with HPAI outbreaks in South Korean duck farms

**DOI:** 10.3389/fvets.2025.1582269

**Published:** 2025-07-03

**Authors:** Saleem Ahmad, Dae Sung Yoo

**Affiliations:** Department of Veterinary Public Health, College of Veterinary Medicine, Chonnam National University, Gwangju, Republic of Korea

**Keywords:** avian influenza, antigen, antibody, hot spots, outbreaks, poultry, surveillance

## Abstract

**Introduction:**

Highly pathogenic avian influenza (HPAI) continues to threaten the poultry industry, particularly in duck farms, where early detection is critical to preventing widespread outbreaks. In South Korea, risk-based antigen and antibody surveillance strategies have been implemented to enhance early warning capabilities. However, the effectiveness of these strategies—especially in terms of testing frequency, timing, and spatial alignment with outbreak risks—remains under-evaluated.

**Methods:**

This study analyzed antigen and antibody surveillance data from South Korean duck farms between 2019 and 2022. Testing frequencies and intervals were assessed across high-risk (October–May) and low-risk (June–September) periods, as well as during non-epidemic (2019–2020) and epidemic (2020–2021 and 2021–2022) seasons. Spatial hotspot analysis (Getis-Ord Gi*) and negative binomial regression were applied to evaluate associations between test patterns and HPAI outbreak occurrence. Additionally, test-to-outbreak intervals were calculated to assess the timeliness of detection.

**Results:**

Antigen testing frequencies were significantly associated with HPAI outbreaks during high-risk periods (coefficient = 0.56, IRR = 1.75, *p* < 0.001). Hot-spot analysis revealed that cold spots received disproportionately more antigen testing than outbreak hotspots (*p* < 0.001), indicating a misalignment in surveillance priorities. Despite intensified testing during epidemic seasons, no significant reductions were observed in the time intervals between the last diagnostic test and outbreak onset (*p* > 0.05), suggesting limited improvement in early detection.

**Discussion:**

The findings highlight both the strengths and limitations of South Korea’s current HPAI surveillance strategy in duck farms. While antigen testing serves as a useful predictor of outbreak risk, the spatial and temporal mismatch between surveillance intensity and actual outbreak distribution undermines its effectiveness. A more adaptive and geographically targeted testing approach is needed to enhance outbreak preparedness and response. These results provide a foundation for optimizing future surveillance strategies to minimize the economic and public health impacts of HPAI.

## 1 Introduction

Systematic documentation of infection cases, infected birds, livestock, and environmental factors is essential for efforts to understand disease epidemiology, detect incursions, monitor incidence, and evaluate the impact of management practices. This process, known as surveillance, involves the systematic collection and analysis of data related to the health statuses of birds and livestock. A surveillance system consists of various measures designed to provide information regarding disease prevalence, incidence, geographic distribution and transmission dynamics within a population (1). This study presents the first comprehensive evaluation of an active serological surveillance system in South Korean duck farms, with the goal of assessing its effectiveness and impact on HPAI prevention through timely detection.

A subtype of the influenza A virus, avian influenza virus (AIV), primarily causes disease in birds. This virus belongs to the Orthomyxoviridae family and comprises 19 hemagglutinin subtypes (H1–H19) and 11 neuraminidase subtypes (N1–N11). In 2022, the highly pathogenic avian influenza (HPAI) H5 virus affected approximately 25 million domestic and wild birds worldwide, leading to an estimated 5.28 million fatalities (2).

HPAI outbreaks in South Korea have been associated with migratory wild birds carrying HPAI viruses (3). Between 2020 and 2022, the number of avian influenza outbreaks in South Korea varied among regions. In 2020, 42 cases were reported, predominantly in Gyeonggi (12 cases), Jeonnam (nine cases), and Chungnam (10 cases), indicating a substantial increase in outbreaks. The highest number of outbreaks occurred in 2021, with 86 cases, primarily in Gyeonggi (25 cases) and Jeonnam (21 cases). Although only 28 outbreaks were reported in 2022, Chungnam (nine cases) and Chungbuk (six cases) remained high-incidence regions (4, 5). The affected areas used in this study can be seen in Supplementary material “Study Areas Maps (.docx).” These patterns highlight the need for targeted surveillance and control measures that consider the dynamic and regionally specific nature of avian influenza epidemics.

Ducks, geese, and other wild, migratory, and free-flying birds constitute important carriers of influenza viruses; they continuously disperse the virus through respiratory fluids and droppings, thereby contributing to its persistence in the environment (6). AIVs have been reported to cross species barriers, spreading from birds to mammals (including humans). Considering the substantial morbidity and mortality associated with human avian influenza infections, this interspecies transmission presents a serious threat to public health. Over the past 20 years, at least six hemagglutinin subtypes of AIVs—H3 (H3N8), H5 (HPAI H5N1, H5N6, and H5N8), H6, H7, H9 (LPAI H9N2), and H10—have demonstrated the ability to infect humans (7). Among these subtypes, H5 and H7 are more important as they are associated with highly pathogenic” (HPAI) strains, whereas H9N2 and H3N8 are considered “low pathogenic” (LPAI) strains but have zoonotic potential of cross species transmission. Due to the zoonotic nature of avian influenza viruses, it is important to strengthen control measures and implement active surveillance systems in birds to prevent potential spread to humans.

In 2008, the South Korean government implemented a nationwide influenza virus surveillance program to detect influenza A viruses in birds that could potentially trigger poultry HPAI outbreaks and pose a risk to human health (8). These existing efforts have primarily concentrated on monitoring migratory and wild birds to track seasonal trends and viral ecology in natural reservoirs (9). While this wildlife-based surveillance has provided valuable insights, it does not adequately assess the effectiveness of surveillance practices within domestic poultry sectors. Our study addresses this gap by focusing specifically on duck farms, which are known amplifiers of HPAI outbreaks. Evaluating the structure and performance of active surveillance in these farms is essential to strengthen early detection and response mechanisms.

Duck farms are receiving increased attention as key sites for HPAI transmission due to the central role of domestic ducks in maintaining and spreading HPAI viruses (10). Efforts to strengthen early detection and control of avian influenza epidemics require a thorough assessment of antigen and antibody testing strategies in duck farm monitoring networks. The sensitivity and timeliness of outbreak detection can be substantially improved through structured and evidence-based surveillance strategies. These include increasing the frequency of testing, ensuring rapid sample collection following risk events, and incorporating both live and dead ducks into routine testing protocols. Such robust surveillance systems enhance the likelihood of early detection before widespread transmission occurs, thereby improving the overall effectiveness of outbreak control measures (11).

The implementation of precise surveillance techniques—defined as targeted, risk-based antigen and antibody testing protocols with optimal frequency and spatial coverage— may reduce the risk of virus transmission while enhancing the monitoring and management of influenza outbreaks. To address this issue, statistical methods and spatial analysis were utilized to examine the relationship between HPAI outbreaks and testing frequencies in South Korean duck farms. The analysis of the surveillance system aims to identify deficiencies or gaps that can be addressed to enhance future surveillance strategies and improve the early detection of HPAI outbreaks.

## 2 Methodology

### 2.1 Data collection

Between 2019 and 2022, a comprehensive dataset was collected to evaluate the performance of HPAI surveillance systems in South Korean duck farms. “The dataset comprising 30,395 individual test records from duck farms, covering the period from 2019 to 2022. These records represent surveillance activities. The data encompass nationwide coverage, including all major poultry-producing provinces in South Korea such as Gyeonggi-do, Jeollabuk-do, Chungcheongnam-do, and Jeju Island which can be observed in the Supplementary material “Study Areas Maps (.docx).” The surveillance involved multiple test types, with the most frequently recorded method being rRT-PCR, used for antigen detection of avian influenza virus in duck populations. The Animal and Plant Quarantine Agency (South Korea) provided surveillance data. These data are available in the Supplementary material as “avian_influenza_surveillance.zip.” One non-epidemic season (2019–2020) and two epidemic seasons (2020–2021, 2021–2022) were analyzed. A 10-meter buffer zone was applied around each duck farm coordinate to define spatial boundaries and prevent overlap with neighboring farms. This ensured that each farm was treated as a distinct spatial unit in the cluster analysis, minimizing the risk of duplicate inclusion or analytical distortion due to farm proximity (12).

Surveillance involved two diagnostic approaches: antibody tests—including ELISA and hemagglutination inhibition (HI)—to detect prior HPAI exposure, and antigen tests—such as rRT-PCR, qRT-PCR, multitube PCR, and egg inoculation—to detect active infections. Testing activities were grouped by two risk periods: high-risk (October–May), and low-risk (June–September) (13). Testing was categorized into two main risk periods: low-risk (June–September) and high-risk (October–May), with the high-risk period aligning with the winter season during which bird migration occurs, potentially facilitating virus dissemination (14).

### 2.2 Descriptive statistics

Descriptive statistics—including maximum, minimum, mean, median, and standard deviation—were computed to assess variations in antigen and antibody test frequencies and time intervals across risk periods (high-risk vs. low-risk) and seasons (epidemic vs. non-epidemic). Each observation represents a farm and period/season combination.

The following metrics were analyzed:

1. Test frequency: number of antigen or antibody tests conducted at a farm during a specific risk period and season.
2. Test interval: number of days between two consecutive antigen or antibody tests at the same farm (Not shown in methodology).
3. Test-to-outbreak interval: number of days between the last test completion date and the subsequent outbreak onset date at the same farm (Not shown in methodology).

Descriptive summaries of these metrics were calculated for each category to evaluate testing intensity, consistency, and responsiveness over time. Stratified analysis by surveillance periods (non-epidemic high-risk, non-epidemic low-risk, epidemic high-risk, epidemic low-risk) provided insights into seasonal and risk-based variations in surveillance implementation.

Maximum (max(X)) and minimum (min(X)) test frequencies for each period (high-risk, low-risk) and season (epidemic, non-epidemic) were calculated using Equation 1, which defines the extrema across all farms within a specified time stratum:


(1) max(X)=max{X1,X2,…,Xn},min(X)=min{X1,X2,…,Xn}

where:

Xi represents the number of antigen or antibody tests conducted for the i-th farm within the specified time stratum, and n is the total number of farms observed in that period.

The mean test frequency was calculated using Equation 2, which computes the average number of tests conducted across all farms within a given time stratum:


(2) $$\bar{X}=\frac{\sum_{i=1}^{n}X_{i}}{n}$$

where:

$\bar{X}$ represents the average number of tests, $X_{i}$ denotes the test frequency for the i-th farm within the specified time stratum, and n is the total number of farms observed in that period.

The standard deviation (SD), quantifying variability in test frequencies, was computed using Equation 3, which measures the dispersion of test counts across farms:


(3) $$\mathrm{SD}=\sqrt{\frac{\sum_{i=1}^{n}{\left(x_{i}-\bar{x}\right)}^{2}}{n-1}}\;$$

where:

SD denotes the standard deviation, $X_{i}$ denotes the test frequency for the i-th farm within the specified time stratum, and n is the total number of farms observed in that period.

### 2.3 Statistical analysis

All statistical tests were two-tailed, with *p*-values less than 0.05 considered statistically significant. In the text, *p*-values were reported using threshold notation only (e.g., *p* < 0.05, *p* > 0.05), with very small values shown as *p* < 0.001. In tables, exact p-values are provided unless very small (e.g., *p* < 0.001) or near the significance threshold (e.g., *p* ≥ or ≤ 0.05), in which case threshold-based reporting is used.

#### 2.3.1 Negative binomial regression analysis for antigen and antibody test frequencies in high-risk and low-risk seasons (epidemic vs. non-epidemic)

To ensure reliable estimation of count-based results, overdispersion in testing frequency data was adjusted for via negative binomial regression, as shown in Equation 4:


(4) $$\begin{array}{l} \log(\mu_{i})=\beta 0+\beta 1(\text{Epidemic Season})+\beta 2(\begin{array}{l} \text{High}-\\ \text{Risk Period} \end{array})+\\ \beta 3(\text{Epidemic Season}\times \text{High}-\text{Risk Period}) \end{array}$$

Where:

μi = expected count of antigen or antibody test frequency.

Dependent variable: Antigen or antibody test frequency per season and risk period.

Independent variables (Categorical Predictors):

- Reference category: Non-Epidemic Season, Low-Risk Period.
- β1 = Epidemic Season (Ref: Non-Epidemic Season).
- β2 = High-Risk Period (Ref: Low-Risk Period).
- β3 = Interaction Term (Epidemic Season × High-Risk Period).

#### 2.3.2 Zero-inflated negative binomial regression analysis for time intervals (days) between consecutive antigen and consecutive antibody tests by season and epidemic status

Time intervals between consecutive antigen and consecutive antibody tests within specific poultry farms were analyzed via zero-inflated negative binomial regression to identify temporal trends in testing patterns. Time intervals were calculated separately for antigen and antibody tests. The unit of interval measurement was “days.” Intervals in days were computed within each individual farm, identified by a unique farm ID and geocoordinates. For each farm, test records were chronologically sorted, and the difference in time between consecutive tests was calculated. To ensure proper estimation of interval days within the same year and period, the high-risk period (October–May) was divided into two distinct phases: October–December and January–May. This temporal division is based on epidemiological evidence of differing outbreak intensity within these sub-periods and winter migratory bird patterns in South Korea, where HPAI outbreaks typically begin between October and December, coinciding with peak waterfowl migration. Dividing the high-risk period into October–December and January–May allows the analysis to reflect seasonal variation in outbreak dynamics and surveillance intensity throughout the migratory and post-migratory seasons, throughout the winter season in South Korea (14). The regression model was defined as:

(i) Count Model (Negative Binomial component): The regression model was defined as shown in Equation 5 for the negative binomial count component, estimating the expected time interval between consecutive antigen or antibody tests:


(5) $$\begin{array}{l} \log(\mu_{i})=\beta 0+\beta 1(\text{Epidemic}-\text{HighRisk}-\text{JanMay})+\\ \beta 2(\text{Epidemic}-\text{LowRisk}-\text{JunSep})+\\ \beta 3(\text{Epidemic}-\text{HighRisk}-\text{OctDec})+\\ \beta 4(\text{NonEpidemic}-\text{LowRisk}-\text{JunSep})+\\ \beta 5(\text{NonEpidemic}-\text{HighRisk}-\text{OctDec})\; \end{array}$$

where:

μ_i_ = the expected interval in days between consecutive antigen/antibody tests for observation *i.*

Dependent variable: Interval (in days) between consecutive antigen and antibody tests.

Independent variables (Predictor Variables):

- Reference category: Non-Epidemic, High-Risk (January–May).
- β1 = Epidemic, High-Risk (January–May).
- β2 = Epidemic, Low-Risk (June–September).
- β3 = Epidemic, High-Risk (October–December).
- β4 = Non-Epidemic, Low-Risk (June–September).
- β5 = Non-Epidemic, High-Risk (October–December).

(ii) Zero-Inflation Model (Logistic component): The corresponding zero-inflation component is defined in Equation 6, modeling the probability of observing a structural zero (i.e., no interval between tests due to repeated or null testing)


(6) $$\begin{array}{l} \text{logit}(\pi_{i})=\gamma 0+\gamma 1(\text{Epidemic}-\text{HighRisk}-\text{JanMay})+\\ \gamma 2(\text{Epidemic}-\text{LowRisk}-\text{JunSep})+\\ \gamma 3(\text{Epidemic}-\text{HighRisk}-\text{OctDec})+\\ \gamma 4(\text{NonEpidemic}-\text{LowRisk}-\text{JunSep})+\\ \gamma 5(\text{NonEpidemic}-\text{HighRisk}-\text{OctDec})\; \end{array}$$

where:

πᵢ: the probability that observation *i* has a structural zero (i.e., an interval of zero days not generated from the count process).

γ0: intercept: log-odds of being a structural zero for the reference category (e.g., Non-Epidemic, High-Risk Jan–May).

γ1: difference in log-odds of being a structural zero when the season is Epidemic–HighRisk–JanMay, compared to the reference.

γ2 to γ5: Effects of other seasonal categories on the probability of structural zero.

#### 2.3.3 Zero-inflated negative binomial regression analysis for intervals between last test completion and onset of infection (2019–2022)

Zero Inflated Negative binomial regression was performed to estimate the time interval between last test completion and outbreak detection for each season. The “last test” refers to the most recent antigen or antibody test (based on test completion date) conducted at the same farm prior to the outbreak occurrence. For each outbreak farm, both antigen and antibody test records were screened, and the test closest in time before the outbreak onset was identified. If multiple test types were conducted, the latest among them was selected. The outbreak date used for interval calculation was the official confirmation date of HPAI, determined through active follow-up testing of all farms within a 3 km radius of the outbreak location. Intervals were computed at the individual farm level. Each test-outbreak pair was required to share identical farm geocoordinates (X, Y) and administrative address. The dependent variable in the model is the interval in days between the last test and the confirmed outbreak date, as calculated using the following model:

(i) Count Model (Negative Binomial Component): The dependent variable in the model is the interval in days between the last test and the confirmed outbreak date, calculated using the negative binomial regression shown in Equation 7:


(7) $$\begin{array}{l} \log(\mu_{i})=\beta 0+\beta 1(\text{Season}\;2020-2021)+\\ \beta 2(\text{Season}\;2021-2022) \end{array}$$

where:

μ_i_ = expected number of days between the last test and outbreak for observation *i.*

Dependent variable: Time interval (in days) between last test completion and the onset of avian influenza outbreaks.

Independent variables (Categorical Predictors):

- Reference category: Season 2019–2020.
- β1 = Season 2020–2021.
- β2 = Season 2021–2022.

(ii) Zero-Inflation Model (Logistic Component): The probability of observing a structural zero — i.e., a 0-day interval between the last test and outbreak onset — was modeled using a logistic regression as presented in Equation 8:


(8) $$\begin{array}{l} \text{logit}(\pi_{i})=\gamma 0+\gamma 1(\text{Season}\;2020-2021)+\\ \gamma 2(\text{Season}\;2021-2022) \end{array}$$

where:

πᵢ: the probability that observation i is a structural zero (i.e., a zero-day interval not explained by random variation).

γ0: intercept (baseline log-odds of a structural zero during Season 2019–2020).

γ1: Change in log-odds of structural zero in Season 2020–2021, relative to 2019–2020.

γ2: Change in log-odds of structural zero in Season 2021–2022, relative to 2019–2020.

#### 2.3.4 Hot-spot analysis

Spatial and temporal analyses were conducted to assess the relationship between county-level testing frequencies and the spatial intensity of HPAI outbreaks. To identify regions with significant clustering of outbreak cases, we applied the Getis-Ord Gi* statistic, as defined in Equation 9:


(9) $$G_{i}^{*}=\frac{\sum_{j=1}^{n}w_{\mathrm{ij}}x_{j}-\bar{x}\sum_{j=1}^{n}w_{\mathrm{ij}}}{\sqrt[{S}]{\frac{\sum_{j=1}^{n}w_{ij}^{2}-{\left(\sum_{j=1}^{n}w_{\mathrm{ij}}\right)}^{2}}{n-1}}}$$

Where:

$x_{j}$ is the value of the attribute for feature j, i.e., the total number of HPAI outbreak cases in county j,

$w_{\mathrm{ij}}$ is the spatial weight between features i and j,

$\bar{x}$ is the mean of the attribute values,

S is the standard deviation of the attribute values used in the Gi* statistic was calculated using Equation 10:


(10) $$S=\sqrt{\frac{\sum_{j=1}^{n}x_{j}^{2}}{n}-{\bar{x}}^{2}}$$

n denotes the total number of features.

The Gi* statistic produces a Z-score (Zi*), which identifies spatial clusters of statistical significance:

- Positive and significant Gi∗ values (*p* ≤ 0.05) indicate hot spots (high-value clustering).
- Negative and significant Gi∗ values (*p* ≤ 0.05) denote cold spots (low-value clustering).
- Non-significant values (*p* > 0.05) illustrate spatial randomness.

Getis-Ord Gi* spatial statistic were applied using the esda. G_Local package (15) in Python programming language version 3.9.21 (16). This method calculates a Z-score (Gi*) for each spatial unit, indicating the degree to which observed values (e.g., number of outbreak cases) are clustered spatially relative to a random distribution. A positive and statistically significant Gi* indicates a hot spot (high-value clustering), while a negative significant Gi* denotes a cold spot (low-value clustering). Non-significant values suggest spatial randomness.

We constructed spatial weights using a k-nearest neighbor matrix with k = 2, which defines the neighborhood of each county. The Gi* statistic relies on assumptions of spatial dependence and requires accurate spatial representation of data points, which we ensured using high-resolution shapefiles and verified outbreak locations from 2003 to 2022. Results were visualized using choropleth maps, and each county was classified into hot spot, cold spot, or non-significant category based on the Z-score distribution. This method is useful for detecting localized outbreak clusters and prioritizing surveillance allocation (17).

#### 2.3.5 Kruskal-Wallis and Dunn’s tests for antigen and antibody tests across hot-spot categories

To assess disparities in testing intensity across different spatial risk zones, we applied the Kruskal-Wallis H test (variance > mean) —a non-parametric method suitable for comparing comparing ranked distributions among more than two groups—to antigen and antibody test frequencies grouped by Gi* categories (hot spot, cold spot, and non-significant). This test assumes independent samples and ordinal or continuous outcome variables (18).

Following significant Kruskal-Wallis results, Dunn’s *post hoc* test was used to identify specific group differences, with Bonferroni adjustment for multiple comparisons. This approach allowed us to determine whether testing frequencies differed systematically based on spatial risk categorization, thereby revealing gaps or concentration in surveillance efforts (19).

#### 2.3.6 Comparison of global and south Korean weekly HPAI outbreak trends (2003–2022)

To investigate broader epidemic patterns, we compared weekly HPAI outbreak trends in South Korea with global trends before and after 2020. We employed the Mann-Kendall trend test, a non-parametric method for detecting monotonic upward or downward trends in time-series data.

This test was selected because it does not require assumptions of linearity or normality, making it suitable for outbreak count data. We calculated Kendall’s Tau coefficient to indicate trend direction and strength, and Sen’s slope to estimate the magnitude of change. This method enabled us to evaluate whether global or national HPAI epidemics were intensifying, stabilizing, or declining across two distinct temporal phases (pre- and post-2020), contributing to understanding how surveillance and control efforts align with changing epidemiological patterns (20).

## 3 Results

### 3.1 Testing frequencies of antigen and antibody tests

Table 1 presents a summary of descriptive statistics for antigen and antibody test frequencies across all seasons and risk periods. Antigen tests exhibited greater variability and were more frequently performed than antibody tests, with increased testing during periods of high epidemic risk (mean: 3.59, maximum: 123 tests). Additionally, testing frequencies remained relatively high during the non-epidemic high-risk period (mean: 3.92), indicating a deliberate emphasis on active infection detection. Conversely, antigen testing was less frequent during the epidemic low-risk period (mean: 1.95), reflecting a lower level of monitoring during these time frames.

**Table 1:** Descriptive statistics of antigen and antibody test frequencies for HPAI detection during epidemic and non-epidemic seasons and high-risk and low-risk periods.

Diagnostic tests	Season	Period	Count/Observations	Mean tests	Median	Std_Dev	Min tests	Max tests
Antibody	Epidemic	High-risk	478	1.90	1	2.61	1	29
	Epidemic	Low-risk	75	1.29	1	0.69	1	4
	Non-epidemic	High-risk	204	1.76	1	1.34	1	15
	Non-epidemic	Low-risk	49	1.12	1	0.33	1	2
Antigen	Epidemic	High-risk	4,140	3.59	2	4.34	1	123
	Epidemic	Low-risk	2,970	1.95	2	1.62	1	36
	Non-epidemic	High-risk	1,693	3.92	3	4.12	1	55
	Non-epidemic	Low-risk	1,183	2.07	2	1.96	1	45
Antigen + Antibody	Epidemic	High-risk	3	1	1	0	1	1
	Non-epidemic	High-risk	13	1	1	0	1	1

Diagnostic Tests: Represents HPAI surveillance testing, including antibody, antigen, or a combination (antigen + antibody) tests. Season: Specifies whether the data correspond to an epidemic or non-epidemic (2019–2020) season. Period: Indicates the testing period is either high-risk (October–May) or low-risk (June–September). Count/Observations: Total number of observations or test instances. Mean Tests: Average number of tests conducted during the specified season and period. Median: Middle value of tests conducted. Std_Dev: Standard deviation, indicating variability in the number of tests conducted. Min Tests and Max Tests: Minimum and maximum number of tests recorded for the group.

Antibody tests were conducted less frequently but with greater consistency, averaging 1.90 tests during the epidemic high-risk period and 1.12 tests during the non-epidemic low-risk period.

### 3.2 Time interval between consecutive antigen tests and consecutive antibody tests

Table 2 presents summaries of the time intervals for antigen and antibody testing, demonstrating that testing during high-risk periods was prioritized in epidemic seasons, whereas a more flexible approach was adopted in non-epidemic periods. Analysis of antigen test intervals revealed a systematic surveillance approach, such that the highest testing frequency occurred during high-risk periods in epidemic seasons. The January–May high-risk period exhibited relatively longer intervals (mean: 26.67 days) with greater variability, whereas the October–December period had the shortest intervals (mean: 17.79 days, median: 13 days), reflecting increased early detection efforts. During low-risk periods (June–September), testing frequency substantially declined; the mean interval increased to 43.23 days, indicating reduced surveillance intensity. A similar pattern was observed during non-epidemic seasons, where testing intervals were slightly longer (mean: 40.51 days), supporting a strategic shift toward lower testing intensity when outbreak risks were minimal.

**Table 2:** Time intervals between consecutive antigen and antibody tests during epidemic and non-epidemic seasons for HPAI surveillance.

Tests	Season	Risk Period	Count/Observations	Mean Interval (Days)	Median	Std_Dev	Min Interval (Days)	Max Interval (Days)
Antigen	Epidemic	Jan-May	5,553	26.67	14	25.32	0	142
		Jun-Sep	2,835	43.23	52	26.98	0	117
		Oct-Dec	3,226	17.79	13	17.88	0	78
	Non-Epidemic	Jan-May	2,324	28.93	16	24.61	0	140
		Jun-Sep	1,274	40.51	49	25.46	0	114
		Oct-Dec	1,680	20.44	14	17.29	0	83
Antibody	Epidemic	Jan-May	152	21.54	7	29.99	0	137
		Jun-Sep	21	25.52	14	28.69	0	87
		Oct-Dec	137	9.19	1	15.28	0	85
	Non-Epidemic	Jan-May	51	37.43	36	28.29	0	100
		Jun-Sep	6	48.33	31	40.47	9	107
		Oct-Dec	13	9.54	10	10.47	0	33

Season: Categorized as epidemic or non-epidemic. Risk Period: Time intervals classified as Jan-May (high-risk), Jun-Sep (low-risk), and Oct-Dec (high-risk). Count/Observations: Total number of observations or tests recorded. Mean Interval (Days): Average number of days between consecutive tests. Median: Middle value in the interval distribution. Std_Dev: Standard deviation, representing variability in test intervals. Min Interval (Days): Shortest recorded interval (in days) between tests. Max Interval (Days): Longest recorded interval (in days) between tests.

For antibody testing, a less frequent but more consistent testing approach was noted. Testing was increased during high-risk epidemic periods; the shortest mean interval (9.19 days) was recorded between October and December, coinciding with the highest outbreak risk. During non-epidemic seasons, antibody test intervals were considerably longer, particularly during the high-risk January–May period (mean: 37.43 days, median: 36 days) and the low-risk June–September period (mean: 48.33 days). These findings indicate a transition from frequent active detection to broader, periodic serological monitoring.

### 3.3 Time intervals (days) between last test completion and onset of outbreaks

**Figure 1:**
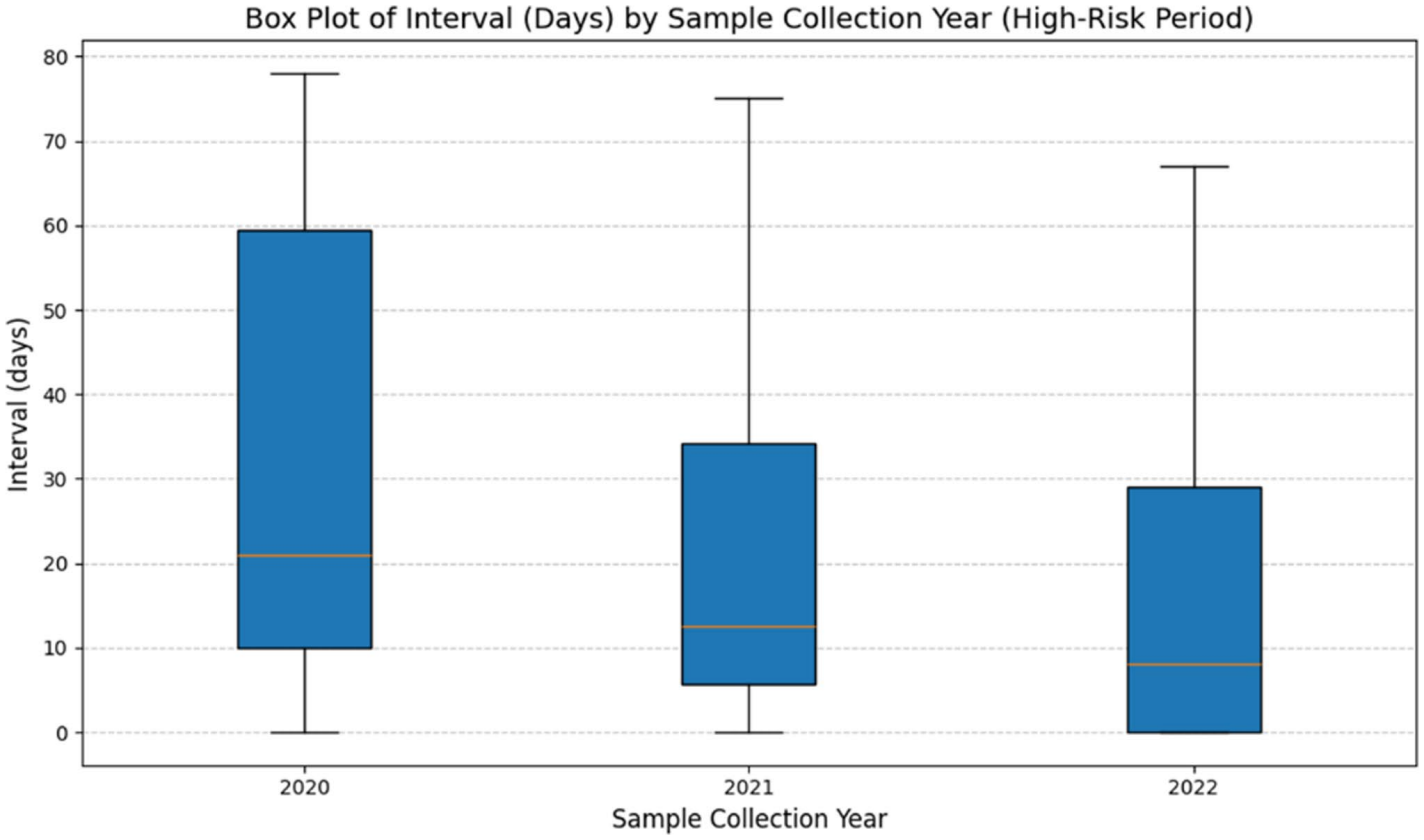
Time intervals between last test completion and onset of infection during each season (2019–2022). This figure presents a box plot illustrating the distribution of intervals (in days) between final test completion date and the onset of outbreaks across three high-risk periods (2020–2022). The horizontal line inside each box represents the median interval, whereas boxes indicate the interquartile range (IQR). The whiskers extend to the minimum and maximum intervals, excluding outliers. The findings show a steady decrease in median intervals over time, suggesting an increased testing frequency. Additionally, the decreasing variability in intervals between 2020 and 2022, as evidenced by compression of the box and whiskers, indicates improved testing consistency during high-risk periods.

Table 3 presents data regarding testing intervals prior to outbreak onset for three seasons (2019–2022), illustrating improvements in surveillance strategies over time. Between 2019 and 2020, 16 outbreaks were reported and testing intervals remained long (mean: 30.92 days, maximum: 78 days), indicating inconsistent surveillance and delayed detection (high variability, SD: 26.23). By 2020–2021, the number of outbreaks increased to 19, whereas testing intervals decreased (mean: 20.64 days, median: 12.5 days, maximum: 75 days), suggesting a more structured testing approach with reduced variability (SD: 19.62). During the 2021–2022 season, testing intervals further shortened (mean: 17.36 days, median: 8 days, maximum: 67 days), coinciding with the highest outbreak count (31 outbreaks). This trend reflects improved reliability and early detection efforts, as indicated by lower variability (SD: 21.11). Figure 1 illustrates the trend of progressively shorter testing intervals during extended outbreak seasons.

**Table 3:** Time intervals between last test completion date and the onset of avian influenza outbreaks.

Season	Period	No. of outbreaks	Mean interval (Days)	Median interval (Days)	Std_Dev	Min interval (Days)	Max interval (Days)	Total observations of intervals (days)
2019–20	High-Risk	16	30.92	21	26.23	0	78	36
2020–21	High-Risk	19	20.64	12.5	19.62	0	75	44
2021–22	High-Risk	31	17.36	8	21.11	0	67	61

Season: Year of observation (2020, 2021, 2022). Period: High-risk period (January–May and October–December). Mean Interval (Days): Average time (in days) between last test and outbreak onset. Median Interval (Days): Middle value of test intervals. Std_Dev: Standard deviation, indicating variability in test intervals; higher values represent greater variation. Min/Max Interval (Days): Shortest and longest intervals observed. Total Observations of Intervals (days): Number of test intervals recorded.

### 3.4 Multivariable regression analysis of antigen and antibody testing frequency

Table 4 presents the results of the regression analysis, which identified significant variations in testing frequency across risk periods. For antigen tests, testing frequency was 1.75 times higher during high-risk periods compared to low-risk periods (IRR: 1.75, *p* < 0.001), indicating significantly intensified surveillance activity during high-risk seasons. However, No statistically significant difference in antigen testing was observed between epidemic and non-epidemic seasons (IRR: 0.95, *p* > 0.05), and the interaction between season and risk period also showed no significant effect (IRR: 0.97, *p* > 0.05), suggesting that increases in testing frequency during high-risk periods were independent of epidemic status.

**Table 4:** Multivariable negative binomial regression results for antigen test frequency evaluation during epidemic and non-epidemic seasons in high-risk and low-risk periods against HPAI outbreaks.

Variables	Coefficient	Std. Error	z-value	*p*-value	IRR	95% CI Lower	95% CI Upper
Season ‘Epidemic’	−0.05	0.04	−1.11	0.26	0.95	−0.13	0.03
Ref: Season ‘Non-epidemic’	1	-	-	-		-	-
Period ‘High-risk’	0.56	0.04	12.80	< 0.001	1.75	0.47	0.64
Ref: Period ‘Low-risk’	1	-	-	-		-	-
Season ‘Epidemic’, Period ‘High-risk’	−0.03	0.05	−0.57	0.57	0.97	−0.13	0.07
Ref: Season ‘Non-epidemic’, Period ‘Low-risk’	1	-	-	-		-	-

Ref, Reference category for categorical variables; IRR, Incident Rate Ratio. This table presents the results of a regression model evaluating the relationship between antigen testing frequency and key factors: season (epidemic vs. non-epidemic) and risk period (high-risk vs. low-risk). Interaction terms between seasons and risk periods showed no significant effect, indicating that testing strategies remained consistent across seasons.

Table 5 shows antibody tests frequency to be 4.35 times higher in high-risk periods (IRR: 4.35, *p* < 0.001), while a modest but marginally statistically significant reduction was observed during epidemic seasons (IRR: 0.71, *p* ≤ 0.05). The interaction term approached marginal significance (IRR: 1.42, *p* ≥ 0.05), suggesting a possible, though not conclusive, season-specific increase in antibody testing during high-risk periods.

**Table 5:** Multivariable negative binomial regression results for antibody test frequency evaluation during epidemic and non-epidemic seasons in high-risk and low-risk periods against HPAI outbreaks.

Variables	Coefficient	Std. error	*z*-value	*p*-value	IRR	95% CI lower	95% CI upper
Season ‘Epidemic’	−0.34	0.17	−1.96	≤ 0.05	0.71	−0.67	−0.00
Ref: Season ‘Non-epidemic’	1	-	-	-		-	-
Period ‘High-risk’	1.47	0.15	9.90	< 0.001	4.35	1.18	1.77
Ref: Period ‘Low-risk’	1	-	-	-		-	-
Season ‘Epidemic’, Period ‘High-risk’	0.35	0.18	1.89	≥ 0.05	1.42	−0.01	0.71
Ref: Season ‘Non-epidemic’, Period ‘Low-risk’	1	-	-	-		-	-

Ref, Reference category for categorical variables; IRR, Incident Rate Ratio. The table shows significantly higher antibody testing frequencies during high-risk periods, whereas seasonal effects had a limited impact.

### 3.5 Zero-inflated negative binomial regression analysis of antigen and antibody test intervals

Table 6 displays the zero-inflated negative binomial regression analysis of antigen test intervals, revealing significant differences between high-risk and low-risk periods, as well as between epidemic and non-epidemic seasons. Antigen test intervals were significantly shorter during epidemic high-risk period (IRR: 0.64, *p* < 0.001 for October–December), indicating increased surveillance for early outbreak detection. In contrast, longer testing intervals were observed during the June–September epidemic low-risk period (IRR: 1.55, *p* < 0.001), suggesting a reduction in surveillance intensity during this time. A similar trend was observed in non-epidemic seasons, where shorter test intervals were recorded during high-risk periods (October–December, IRR: 0.73, *p* < 0.001) and longer intervals were detected during low-risk periods (June–September, IRR: 0.73, *p* < 0.001). These findings suggest that proactive surveillance efforts were maintained even in non-epidemic years.

**Table 6:** Multivariable zero-inflated negative binomial regression for evaluating time intervals between consecutive antigen tests during epidemic and non-epidemic seasons in high-risk and low-risk periods against HPAI outbreaks.

Variables	Coefficient	Std. error	*z*-value	*p*-value	IRR	95% CI lower	95% CI upper
inflate_const	−3.32	0.13	−26.18	0	0.04	−3.57	−3.07
inflate_Epidemic- Jan-May	1.04	0.14	7.66	0	2.83	0.77	1.3
inflate_Epidemic- Jun-Sep	0.79	0.15	5.38	0	2.2	0.5	1.08
inflate_Epidemic- Oct-Dec	0.66	0.15	4.44	0	1.93	0.37	0.96
inflate_Non-Epidemic- Jun to Sep	0.3	0.19	1.57	0.12	1.35	−0.07	0.67
inflate_Non-Epidemic- Oct to Dec	0.71	0.16	4.29	0	2.03	0.38	1.03
const	3.4	0.02	197.02	0	29.96	3.37	3.43
(Season ‘Epidemic’- Period ‘High-risk’) (Jan-May)	−0.02	0.02	−0.94	0.35	0.98	−0.06	0.02
(Ref: Season ‘Non-Epidemic’- Period ‘High-risk’) (Jan-May)	1	-	-	-	-	-	-
(Season ‘Epidemic- Period ‘Low-risk’) (Jun-Sep)	0.44	0.02	18.95	< 0.001	1.55	0.4	0.49
(Ref: Season ‘Non-Epidemic’- Period) ‘High-risk’ (Jan-May)	1	-	-	-	-	-	-
Season ‘Epidemic’- Period ‘High-risk’ (Oct-Dec)	−0.45	0.02	−19.75	< 0.001	0.64	−0.5	−0.41
(Ref: Season ‘Non-Epidemic’- Period ‘High-risk’) (Jan-May)	1	-	-	-	-	-	-
Season ‘Non-Epidemic’- Period ‘Low-risk’ (Jun-Sep)	0.35	0.03	12.06	< 0.001	1.42	0.29	0.41
(Ref: Season ‘Non-Epidemic’- Period ‘High-risk’) (Jan-May)	1	-	-	-	-	-	-
Season ‘Non-Epidemic’- Period ‘High-risk’ (Oct-Dec)	−0.31	0.03	−11.5	< 0.001	0.73	−0.36	−0.26
(Ref: Season ‘Non-Epidemic’- Period ‘High-risk’) (Jan-May)	1	-	-	-	-	-	-
alpha	0.63	0.01	79.28	< 0.001	1.88	0.61	0.64

Ref, Reference category for categorical variables; IRR, Incident Rate Ratio. The table presents strategic testing intervals, emphasizing shorter intervals during high-risk period “Oct-Dec” during epidemic season.

Table 7 shows a similar pattern for antibody test intervals, with increased surveillance during epidemic and non-epidemic high-risk periods. Antibody test intervals were significantly shorter from October to December (IRR: 0.37, *p* < 0.001), reflecting enhanced serological surveillance for early viral circulation detection. The June–September low-risk period (IRR: 0.95, *p* > 0.05) showed no significant change, suggesting that antibody testing was not prioritized when outbreak risks were minimal. The shortest antibody test intervals were recorded during the October–December high-risk period highlighting the importance of early outbreak detection. Although the results were not statistically significant, testing frequency remained moderate in non-epidemic seasons (IRR: 1.15, *p* > 0.05); shorter intervals were observed during the high-risk period.

**Table 7:** Multivariable zero-inflated negative binomial regression for evaluating time intervals between consecutive antibody tests during epidemic and non-epidemic seasons in high-risk and low-risk periods.

Variables	Coefficient	Std. error	*z*-value	IRR	*p*-value	95% CI lower	95% CI upper
inflate_const	−2.15	0.55	−3.9	0.12	0	−3.23	−1.07
inflate_Epidemic- Jan-May	1.14	0.58	1.97	3.13	0.05	0	2.27
inflate_Epidemic- Jun-Sep	1.56	0.73	2.14	4.76	0.03	0.14	2.99
inflate_Epidemic- Oct-Dec	1.76	0.58	3.05	5.81	0	0.63	2.89
inflate_Non-Epidemic- Jun to Sep	−9.41	132.47	−0.07	0	0.94	−269.04	250.22
inflate_Non-Epidemic- Oct to Dec	1.42	0.87	1.63	4.14	0.1	−0.29	3.13
const	3.73	0.17	22.45	41.68	0	3.41	4.06
(Season ‘Epidemic’- Period ‘High-risk’) (Jan-May)	−0.35	0.2	−1.79	0.7	0.07	−0.74	0.03
(Ref: Season ‘Non-Epidemic’- Period ‘High-risk’) (Jan-May)	1	-	-	2.72	-	-	-
(Season ‘Epidemic- Period ‘Low-risk’) (Jun-Sep)	−0.05	0.35	−0.15	0.95	0.88	−0.73	0.63
(Ref: Season ‘Non-Epidemic’- Period ‘High-risk’) (Jan-May)	1	-	-	2.72	-	-	-
Season ‘Epidemic’- Period ‘High-risk’ (Oct-Dec)	−1	0.21	−4.75	0.37	< 0.001	−1.41	−0.59
(Ref: Season ‘Non-Epidemic’- Period ‘High-risk’) (Jan-May)	1	-	-	2.72	-	-	-
Season ‘Non-Epidemic’- Period ‘Low-risk’ (Jun-Sep)	0.14	0.48	0.3	1.15	0.76	−0.8	1.08
(Ref: Season ‘Non-Epidemic’- Period ‘High-risk’) (Jan-May)	1	-	-	2.72	-	-	-
Season ‘Non-Epidemic’- Period ‘High-risk’ (Oct-Dec)	−1.09	0.43	−2.53	0.34	0.01	−1.92	−0.25
(Ref: Season ‘Non-Epidemic’- Period ‘High-risk’) (Jan-May)	1	-	-	2.72	-	-	-
alpha	1.19	0.14	8.27	3.29	0	0.91	1.48

Ref, Reference category for categorical variables; IRR, Incident Rate Ratio. The table presents antibody test intervals for epidemic and non-epidemic seasons, highlighting shorter intervals during high-risk periods in epidemic and non-epidemic years, particularly from October to December.

### 3.6 Zero-inflated negative binomial regression for time intervals between last test completion and onset of avian influenza outbreaks

Table 8 shows the results of zero-inflated negative binomial regression analysis, which showed no significant reduction in time intervals between last tests and avian influenza outbreaks over the study period, indicating inactive early detection. The 2021–2022 season had a slight, non-significant decrease compared to 2019–2020 (IRR: 0.80, *p* > 0.05), and also 2020–2021 showed no active testing intensity (IRR: 0.70, *p* > 0.05). Despite intensified testing, outbreak detection timeliness has not advanced. More frequent, strategically timed tests and real-time data integration are needed for better surveillance.

**Table 8:** Zero-inflated negative binomial regression for time intervals between last test completion date and the onset of avian influenza outbreaks.

Variables	Coefficient	Std. Error	z-value	P-value	IRR	95% CI Lower	95% CI Upper
inflate_Intercept	−2.15	0.57	−3.79	0	0.12	−3.26	−1.04
inflate_ Season-‘2020–2021’	0.4	0.72	0.56	0.58	1.49	−1.01	1.81
inflate_ Season-‘2021–2022’	1.62	0.63	2.58	0.01	5.05	0.39	2.85
Intercept	3.54	0.15	24.28	0	34.47	3.26	3.83
Season-‘2020–2021’	−0.35	0.2	−1.77	0.08	0.7	−0.75	0.04
Ref: Season-‘2019–2020’	1	-	-	-	-	-	-
Season-‘2021–2022’	−0.22	0.2	−1.13	0.26	0.8	−0.61	0.16
Ref: Season-‘2019–2020’	1	-	-	-	-	-	-
alpha	0.65	0.1	6.47	< 0.001	1.92	0.45	0.85

Ref, Reference category for categorical variables; IRR, Incident Rate Ratio. This table presents zero-inflated negative binomial regression findings for time intervals that did not significantly decrease across epidemic seasons. The analysis compares the duration between last test completion and the onset of avian influenza outbreaks in the 2020–2021 and 2021–2022 seasons, relative to 2019–2020.

### 3.7 Spatial analysis of HPAI outbreaks and testing frequencies

**Figure 2:**
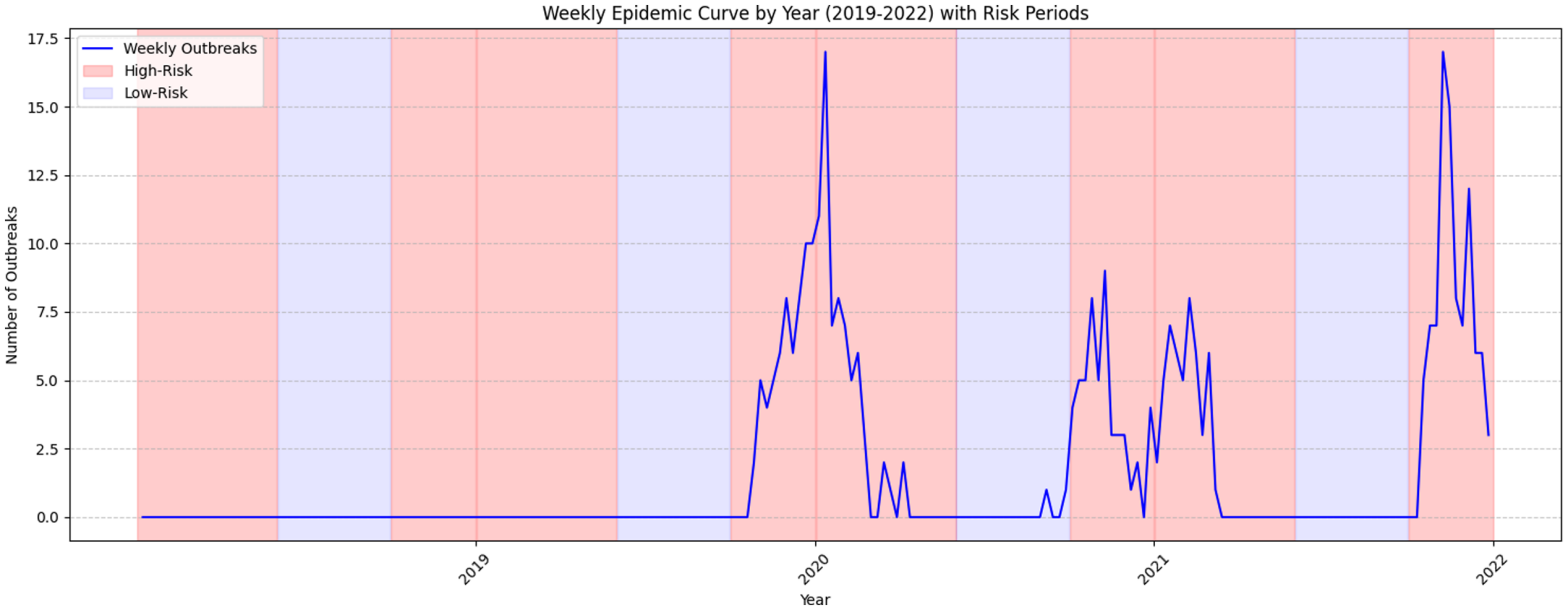
Distribution and progression of HPAI outbreak cases across epidemic seasons (2019–2022). This epidemic curve illustrates the distribution and progression of HPAI outbreak cases spanning epidemic seasons from 2019 to 2022. The figure provides insights into outbreak dynamics and trends during high-risk and low-risk periods.

**Figure 3:**
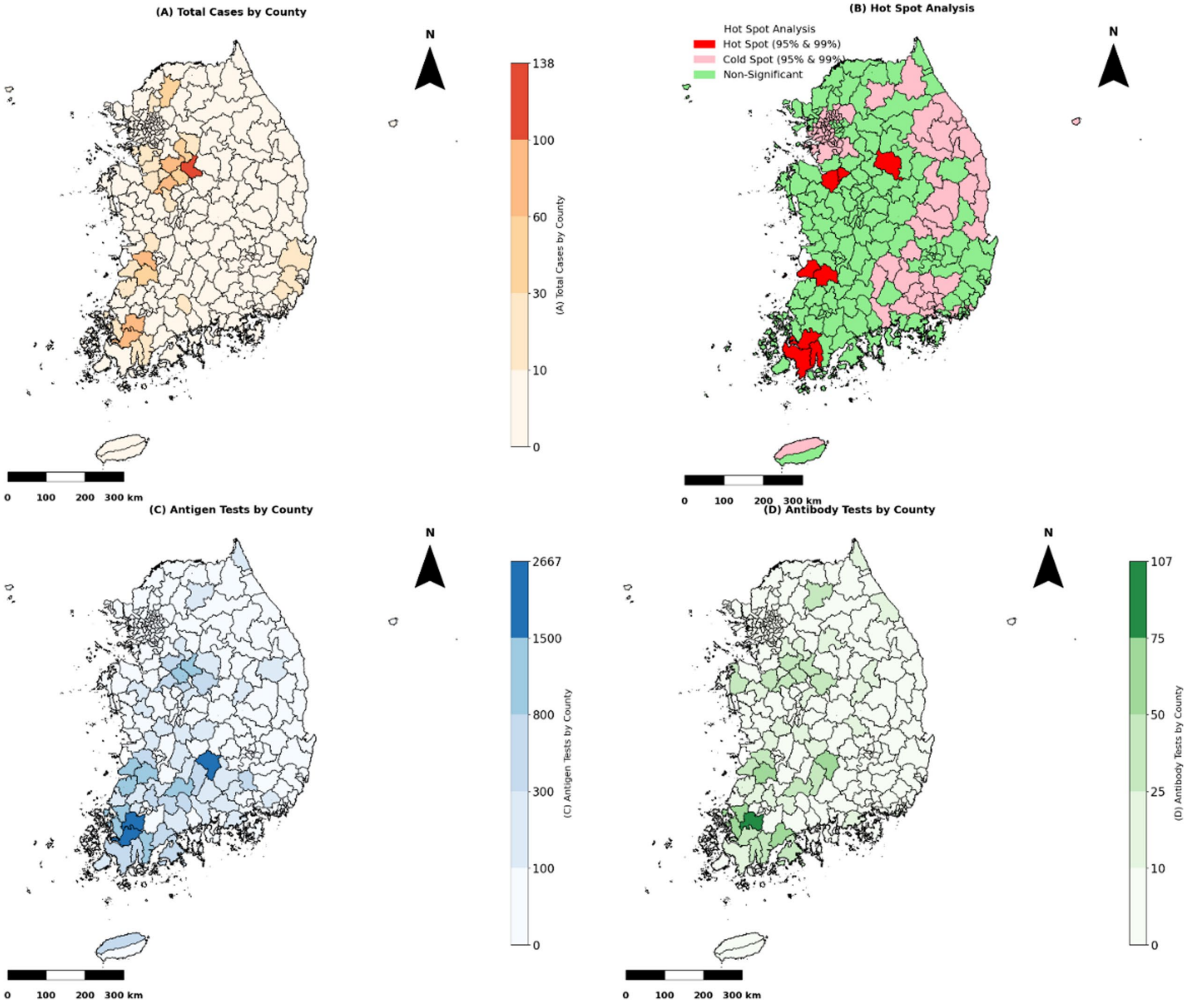
Spatial analysis of HPAI outbreak cases and testing frequencies. “Note: Test frequencies are presented as absolute counts per county and are not adjusted for poultry population size or farm density, which may vary regionally and affect interpretation”. **(A)** The total number of HPAI epidemic cases by county (2003–2022) is represented as a choropleth map. Counties with more severe outbreaks are shaded darker to emphasize higher case concentrations. **(B)** Getis-Ord Gi* hot spot analysis identified spatial clusters of HPAI outbreaks. Cold spots (pink) indicate significant clustering of low outbreak cases at 95 and 99% confidence levels, non-significant areas (green) show no clustering, and hot spots (red) indicate areas with substantial clustering of high epidemic cases at 95 and 99% confidence levels. **(C)** Antigen test distribution by county. Counties with higher antigen test frequencies are shaded in darker blue, indicating priority areas for active surveillance. **(D)** Antibody test distribution by county. Counties with higher antibody test frequencies are shaded in darker green, usually indicating where post-outbreak surveillance is emphasized.

Figure 2 illustrates the spatial distribution of HPAI outbreaks from 2019 to 2022. Variations in testing patterns were identified across different regions based on the geographic distribution of outbreaks and testing frequencies. Figure 3 provides a detailed spatial analysis: (A) depicts the number of outbreak cases at the county level and (B) presents the spatial clustering of outbreaks using Getis-Ord Gi* analysis, categorizing counties into hot spots, cold spots, and non-significant risk areas. Moreover, panels (C) and (D) display the spatial distributions of antigen and antibody testing frequencies across counties, respectively.

Table 9 lists descriptive statistics indicating that antigen testing was substantially higher in cold spots (mean: 4.2) than in hot spots (mean: 2.6), whereas antibody test frequencies were slightly higher in non-significant risk areas (mean: 1.8). Testing frequencies remained moderate in non-significant zones. The Kruskal-Wallis test confirmed significant differences in antigen test frequencies among hot-spot categories (*p* < 0.001), whereas no significant differences were detected for antibody test frequencies (*p* > 0.05). Dunn’s *post hoc* test revealed substantial differences in antigen test frequencies between cold spots and other zones; antibody test frequencies in hot spots, cold spots, and non-significant areas did not significantly differ.

**Table 9:** Descriptive statistics for antigen and antibody tests in hotspot categories.

Tests	Hotspot_type	Count	Mean	Std	Min	25%	50%	75%	Max
Antigen	Cold	1161	4.20	6.02	1	1	2	5	123
	Hot	2049	2.64	2.23	1	1	2	3	18
	Non-significant	6772	2.88	3.25	1	1	2	3	68
Antibody	Cold	149	1.54	1.20	1	1	1	2	11
	Hot	139	1.78	1.94	1	1	1	2	15
	Non-significant	518	1.82	2.39	1	1	1	2	29

Std, Standard deviation; Min, Minimum value of antigen or antibody tests; 25, 50, 75%: First quartile, median (second quartile), and third quartile values of antigen or antibody tests; Max, Maximum value of antigen or antibody tests. This table provides descriptive statistics for antigen and antibody tests across different hotspot categories (hot spots, cold spots, and non-significant areas). It summarizes the distribution of test counts, mean values, and variability within each category.

Table 10 presents the spatial distribution and statistical variations in antigen testing across surveillance regions. The Dunn’s post hoc test builds on this categorization and demonstrates that antigen testing frequencies were significantly higher in cold spots than in both hot spots and non-significant zones. This grouping adds value by highlighting a potential misalignment between surveillance intensity and outbreak risk, as higher testing occurred in cold spots (areas with historically low outbreak clustering). This finding suggests a possible misallocation of surveillance resources and reinforces the importance of spatially risk-adjusted surveillance strategies.

**Table 10:** Dunn’s test for significant differences between hotspot Gi categories based on antigen tests.

	Cold spot	Hot spot	Non-significant	Group
Cold spot	1	<0.001	<0.001	A
Hot spot	<0.001	1	1	B
Non-significant	<0.001	1	1	B

This table presents statistically significant differences between Group A and Group B based on the correlation between Gi* categories. Gi* categories from distinct groups exhibit statistically significant differences in antigen testing, whereas Gi* categories within the same group show no significant variation in antigen tests.

### 3.8 Statistical test for south Korean’s HPAI epidemic trends compared to global HPAI outbreaks trends

**Figure 4:**
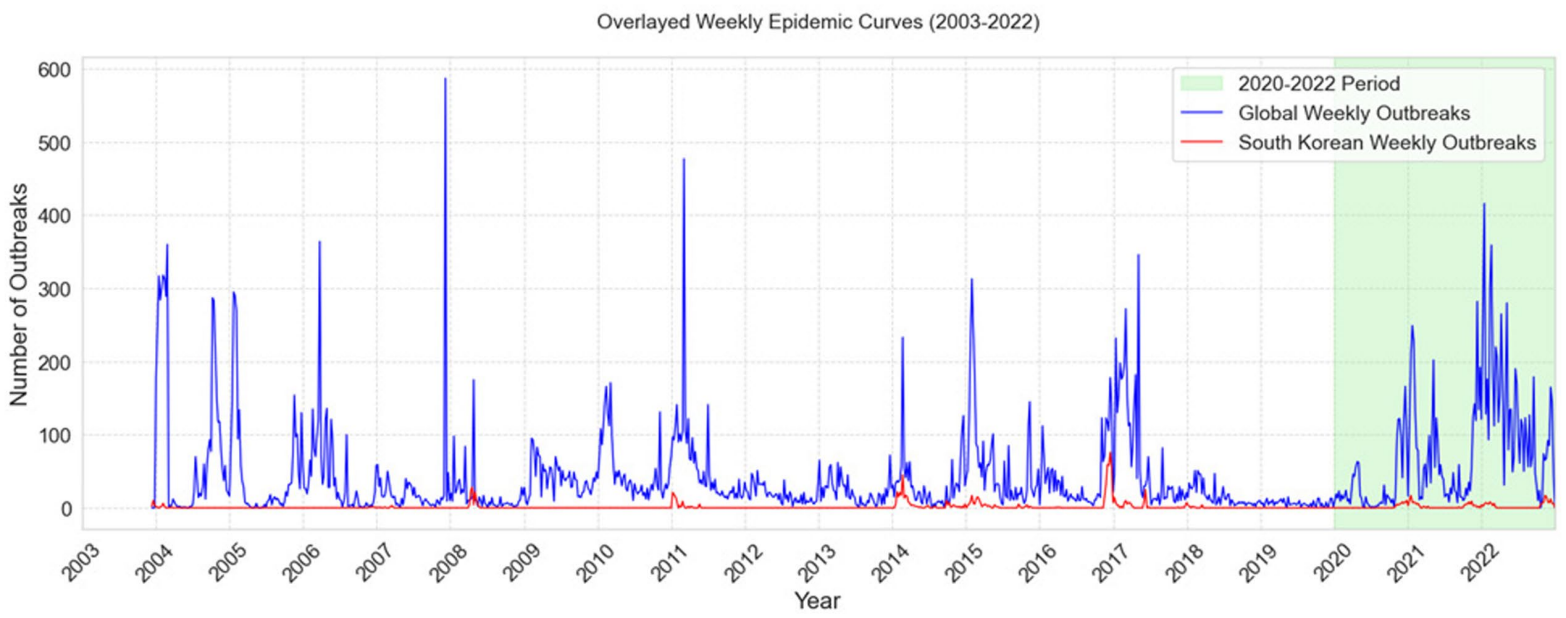
Comparison of Global and South Korean Weekly HPAI Outbreak Trends (2003–2022). The figure depicts overlayed weekly epidemiological curves (2003–2022) of global avian influenza outbreaks (blue) and South Korean outbreaks (red), along with the 2020–2022 timeframe indicated in green.

Figure 4 represents the South Korean epidemic curve in red, allowing for an assessment of the degree of severity of highly pathogenic avian influenza (HPAI) outbreaks before and after 2020 to the corresponding global epidemics, shown by a blue curve. The Global epidemic data was obtained from “MAFRA,” the Ministry of Agriculture, Food and Rural Affairs of South Korea, and “FAO,” the Food and Agriculture Organization of the United Nations (5).

Table 11 presents Mann Kendall trends tests analysis which confirmed a worsening global trend in HPAI outbreaks, showing a declining trend before 2020 (*p* < 0.05, Tau = −0.07) but a rapid rise after 2020 (*p* < 0.001, Tau = 0.31, slope = 0.49 outbreaks per week).

**Table 11:** Mann-Kendall trend test for HPAI epidemics in South Korea and global.

HPAI Outbreak Trends	Period	Trend	p-value	Z-score	Kendall’s Tau	S-statistic	Variance (Var S)	Slope	Intercept
Global weekly Epidemic Curve	Before 2020	Decreasing	0.0031	−2.96	−0.07	−23949	65472989.67	−0.008	22.98
	After 2020	Increasing	< 0.001	5.73	0.31	3813	442202.33	0.49	10.31
South Korea weekly Epidemic Curve	Before 2020	Increasing	< 0.001	4.00	0.06	20985	27466465.67	0	0
	After 2020	Increasing	0.002	3.08	0.14	1748	322464	0	0

This table confirms patterns of HPAI epidemics before and after 2020, with statistical measures for global and South Korean weekly epidemic curves. HPAI outbreaks before and after 2020.

The epidemic trend in South Korea, on the other hand, grew slightly but remained stable. Both before and after 2020, there was a substantial rising trend (*p* < 0.001, Tau = 0.06 and p < 0.05, Tau = 0.14), but the slope was zero, suggesting that the frequency of outbreaks remained consistent over time.

## 4 Discussion

Significant variations in testing frequencies during epidemic and non-epidemic seasons, as well as high-risk and low-risk periods, were evident based on descriptive statistics of HPAI surveillance testing. In both epidemic and non-epidemic seasons, antigen testing demonstrated higher mean frequencies and greater variability than antibody testing. There were only a few farms where antigen and antibody tests were used in combination, resource allocation along with logistical challenges may have hindered the simultaneous use of both tests. These combined antigen and antibody tests were not used in further analysis. To assess the effects of seasonality and risk periods on testing activity, we performed multivariable negative binomial regression analyses, interpreting results using incident rate ratios (IRRs) for both antigen and antibody test frequencies. For antigen testing, the frequency was significantly higher during high-risk periods, with an IRR of 1.75 (*p* < 0.001), indicating more frequent testing during October to May when avian influenza transmission risk is elevated. However, the difference between epidemic and non-epidemic seasons was not statistically significant (IRR: 0.95, *p* > 0.05), suggesting testing efforts remained relatively consistent regardless of whether a large-scale outbreak was underway. The interaction between season and risk period was also non-significant (IRR: 0.97, *p* > 0.05), reinforcing the interpretation that risk-period timing, rather than epidemic status, guided test frequency decisions. While antibody testing frequency was significantly higher during high-risk periods overall (IRR = 4.35, *p* < 0.001), the interaction between season and risk period was not statistically significant (IRR = 1.42, *p* ≥ 0.05). This suggests that the increase in antibody testing was likely driven by the risk period itself rather than being specifically amplified during epidemic seasons. Although the point estimate indicates a potential increase in antibody testing during high-risk epidemic phases, the lack of statistical significance limits strong inferences. These findings highlight a possible gap in the targeted intensification of antibody surveillance during epidemic periods. Future strategies should aim to integrate dynamic risk indicators—such as outbreak proximity, species-specific vulnerability, and historical infection status—to ensure antibody testing is more precisely aligned with real-time epidemic risk, particularly in high-density farming regions (21).

The present findings demonstrate how surveillance initiatives can be tailored to various risk levels, illustrating the system’s capacity for real-time adaptation to evolving epidemiological conditions. When a risk-responsive monitoring approach is utilized, testing frequencies, resource allocations, and intervention strategies are proportionately adjusted based on regions and seasons with increased epidemic likelihood. The prioritization of high-risk areas and maintenance of baseline monitoring in lower-risk areas enhances early detection capabilities, limits disease spread, and optimizes surveillance efficiency. During the management of avian influenza, where risk factors such as migratory bird patterns, farm density, and environmental conditions continuously fluctuate, a data-driven and adaptive framework is particularly important. This approach strengthens disease control efforts and supports a more proactive and cost-effective strategy for preventing potential outbreaks by aligning surveillance intensity with epidemiological risk.

Failure to detect the virus before it spreads, particularly during high-risk periods, permits transmission by duck species that exhibit no clinical signs, allowing the virus to spread to nearby poultry farms and wild bird populations (22). Studies of avian influenza outbreaks have shown that even asymptomatic domestic ducks can continuously shed the virus from the cloaca and oral cavity, contaminating the environment and posing a risk to other poultry and potentially to humans (23, 24). These studies underscore the need for robust active surveillance strategies with optimal testing that extend beyond the detection of visible symptoms. A comprehensive avian influenza surveillance system should integrate both antigen and antibody testing to enhance monitoring and disease control. Based on our findings, antigen tests should be increased in epidemic or high-risk season which may detect the presence of viral proteins, enabling identification of active infections before the virus dissemination while maintaining routine antibody testing year-round to examine seroconversion trends. Antibody tests detect prior exposure or infection by characterizing the host’s immune response to the virus. This information is particularly valuable for efforts to understand the virus’s epidemiology, assess its spread within a population, and evaluate the effectiveness of vaccination campaigns. By incorporating both antigen and antibody testing, surveillance systems can achieve increasingly accurate and timely understanding of avian influenza dynamics, facilitating more effective disease management strategies (25). Furthermore, incorporating farm-specific risk assessments and adjusting testing intervals based on epidemiological patterns may boost resource allocation and early preventive efforts.

Analyses of time intervals between consecutive antigen and consecutive antibody tests provide insights into temporal surveillance strategies during epidemic and non-epidemic seasons. For antigen tests, significantly shorter intervals were observed during epidemic high-risk months (October–December) compared to the reference non-epidemic high-risk period (IRR: 0.64, *p* < 0.001), indicating intensified surveillance efforts during peak risk periods. In contrast, low-risk months showed longer intervals, aligning with lower perceived risk. For antibody tests, the shortest intervals were also recorded during epidemic high-risk months (IRR: 0.37, *p* < 0.001), supporting the hypothesis of enhanced serological monitoring when outbreak risk is elevated. However, other comparisons, such as epidemic low-risk periods, did not yield statistically significant changes (IRR: 0.95, *p* > 0.05), suggesting no notable adjustment in testing strategy during these months. These results, derived from zero-inflated negative binomial regression, support the strategic deployment of testing resources in response to seasonal and epidemiological risk, and highlight the adaptability of surveillance systems to dynamic outbreak threats. A more comprehensive assessment of HPAI monitoring strategies could be achieved by incorporating findings from a study that evaluated multiple detection methods among vaccinated duck flocks in France using mathematical models. In that study, enhanced passive surveillance—specifically, the weekly inspection of dead birds—was regarded as the most effective strategy for outbreak detection. Conversely, monthly testing of live birds was considered less effective. These studies underscore the importance of an integrated monitoring approach that incorporates both antigen and antibody testing, extending beyond routine live bird testing to include systematic surveillance of dead birds (11). A comprehensive approach to HPAI detection and control is supported by the integration of findings regarding active surveillance testing frequencies with enhanced passive surveillance techniques, as indicated in previous studies. This combined strategy can strengthen early detection capacity and facilitate more effective responses to potential outbreaks.

Analyses of time intervals between the last test completion date and the onset of avian influenza outbreaks offer important insight into the timeliness of current surveillance strategies. Zero-inflated negative binomial regression revealed that these intervals did not significantly decrease over the study period, suggesting limited improvement in early detection capacity. Specifically, while the 2021–2022 season had a slightly lower interval than 2019–2020 (IRR: 0.80, *p* > 0.05), this difference was not statistically significant. Similarly, the 2020–2021 season also showed no meaningful reduction (IRR: 0.70, *p* > 0.05). These findings indicate that, despite intensified testing efforts during outbreak years, the timeliness of outbreak detection has not demonstrably improved across seasons. The results underscore a need for enhancing surveillance responsiveness through more targeted and timely testing protocols, ideally supported by real-time risk indicators and adaptive scheduling. Factors such as flock transitions and the associated downtime between production cycles may partially explain the observed prolongation of the interval between last test completion and the onset of avian influenza outbreaks. This transitional phase, commonly referred to as the “down period,” may contribute to decreased testing frequency, potentially delaying outbreak detection. Such lapses in surveillance systems highlight the importance of strategic testing protocols that consider farm management practices to ensure continuous monitoring and early identification of infections. Early detection is essential to limit the spread of avian influenza within poultry populations and prevent potential zoonotic transmission. There is evidence that improved monitoring techniques, including increased sampling frequency, enhance the early detection of low pathogenic avian influenza. However, the sustainability of excessively frequent sampling remains a concern; there is a need for balanced strategies that increase surveillance during high-risk periods or after initial detection of an outbreak in duck populations (26, 27).

Examination of the spatial distributions of antigen and antibody test frequencies across various hotspot categories revealed distinct patterns in testing intensity and outbreak clustering. Hot spots exhibited considerably lower antigen test frequencies (mean = 2.63, max = 18), whereas cold spot regions had the highest antigen screening rates (mean = 4.20, max = 123). The Kruskal-Wallis test confirmed significant differences in antigen test frequencies among hotspot categories (*p* < 0.001). This finding was supported by Dunn’s *post hoc* test, which indicated that antigen testing rates were significantly higher in cold spot areas than in hot spot or non-significant areas. Conversely, no significant differences were observed in antibody test rates between hotspot categories (*p* > 0.05), suggesting that antibody testing was uniformly distributed regardless of outbreak severity. The enhancement of surveillance system strategies through implementation of risk-based, robust testing both spatially and temporally could improve early detection of avian influenza and help to prevent future outbreaks (28).

These spatial findings highlight that current surveillance resources may be disproportionately allocated to areas with historically lower outbreak intensity (cold spots), while high-risk hot spot areas received relatively fewer antigen tests. This emphasizes the need for spatial targeting in future surveillance frameworks to ensure testing is aligned with actual outbreak clustering.

Evaluating the surveillance system in South Korea in the broader sense, HPAI outbreak trends showed distinctive variations between the global and South Korean epidemic curves. Globally, the Mann-Kendall trend test showed a substantial shift from a dropping trend before to 2020 (Tau = −0.07, *p* < 0.05) to a rapid increase post-2020 (Tau = 0.31, *p* < 0.001, slope = 0.49 outbreaks/week), as seen in Figure 4. This implied a significant increase in HPAI severity globally beyond 2020, most likely due to ecological or anthropogenic reasons. In contrast, South Korea showed a constant, if slightly rising, trend across both time periods (Tau = 0.06, *p* < 0.001 before 2020; Tau = 0.14, *p* < 0.05 after 2020). The slope was zero, showing no significant change in outbreak frequency. The results showed that while South Korea remained relatively stable, HPAI outbreaks showed sharp global rising pattern demonstrating the crucial role of surveillance and control initiatives in managing outbreaks.

The use of the Mann-Kendall test strengthened this interpretation by offering formal statistical evidence that complements visual observations (Figure 4). It helped distinguish between apparent fluctuations and true directional trends, thereby supporting the urgency for more robust surveillance and early-warning mechanisms globally and nationally.

The South Korean avian influenza surveillance system in operation between 2014 and 2018 primarily relied on environmental sampling of fecal matter from migratory bird stopover sites to detect circulating influenza A viruses and assess their genetic diversity (9). This approach provided insights into the presence of low-pathogenic avian influenza viruses and their role in the transmission cycle. However, it had several limitations, particularly in terms of targeted procedures for high-risk poultry farming zones and real-time outbreak monitoring. In contrast, the current surveillance system evaluation incorporates both antigen and antibody testing in duck farms, rather than predominantly relying on environmental sampling of wild birds. This antigen/antibody testing strategy enables identification of both recent viral exposure (through antibody testing) and active infections (through antigen testing).

The assessment explored in this study is particularly important because previous evaluations of surveillance systems primarily focused on characterizing virus prevalence in wild birds, limiting their ability to track and assess ongoing outbreaks at the farm level. In summary, the integration of spatial risk-based testing, quantitative surveillance interval assessments, and active farm-level monitoring represents a major advancement in avian influenza surveillance strategies. By incorporating both spatial and temporal risk evaluations, this improved system enables a more proactive and data-driven response compared with earlier surveillance methods, which largely relied on passive monitoring and wild bird sampling. These improvements optimize resource allocation and enhance early detection capabilities by systematically addressing key deficiencies in previous monitoring approaches, such as inadequate outbreak tracking at the farm level, irregular testing frequencies, and limited spatial coverage. Consequently, this enhanced surveillance system strengthens South Korea’s preparedness for avian influenza and facilitates a more efficient and evidence-based response to potential outbreaks.

Despite substantial advancements in the understanding and management of avian influenza outbreaks, multiple challenges remain. Continued research, sustained monitoring, and the application of modern technologies such as artificial intelligence and machine learning are essential to improve knowledge of the virus’s evolutionary potential, transmission dynamics, and host–pathogen interactions. Integrating AI-driven risk assessment models, digital diagnostic devices, and real-time genome sequencing in surveillance system may assist with early detection and outbreak prevention. Integrating spatial analysis with AI and genome sequencing may ensure that outbreaks can be detected and promptly addressed through optimal testing strategies in high risk predicted areas and at-risk species (29–32). Furthermore, collaborative research and ongoing monitoring remain critical for advancing the understanding of host–pathogen interactions, ecological risk factors, and viral transmission patterns. Future surveillance frameworks can achieve greater efficacy, accuracy, and adaptability by integrating these innovations, thus ensuring timely interventions that mitigate the impacts of avian influenza on public health and the poultry industry.

### 4.1 Study limitations

Although this study identified substantial advancements in evaluating the HPAI serological surveillance system across South Korean duck farms, some limitations must be acknowledged. First, as part of control measures, poultry farms within a 3-km radius of an outbreak site are either depopulated or culled during epidemic periods (33). This practice affects the surveillance system by reducing the number of farms available for ongoing testing, which decreases overall test frequency and may lead to underestimation of testing intensity in high-risk areas. The exclusion of these farms from active surveillance during culling periods may also hinder the detection of residual viral transmission in neighboring farms, resulting in less precise assessments of test frequency. While we acknowledged the impact of this on surveillance coverage, we did not adjust test intervals during periods when farms were non-operational due to depopulation. As a result, test frequency estimates may not fully reflect surveillance gaps caused by farm unavailability. This was primarily due to the lack of consistently available metadata specifying exact periods of farm inactivity or reactivation. Future studies should consider incorporating time-varying exposure indicators to account for periods when farms are inactive or removed from the surveillance pool, thereby improving the accuracy of surveillance intensity assessments and interval modeling.

Second, the reallocation of new duck flocks may contribute to underestimation of the efficacy of early detection measures, particularly regarding analyses of time intervals between last test completion and the onset of infection. When new flocks are introduced during farm operation down periods, a temporal gap in testing occurs, potentially leading to miscalculation of testing efficiency. Recently introduced flocks may not undergo immediate and comprehensive testing, which increases the risk of undetected viral infections and may prolong HPAI transmission.

The interpretation of antigen and antibody test frequencies across counties was based on absolute test counts without normalization for poultry population size or the number of duck farms. Due to the unavailability of county-level data on bird populations or farm counts during the study period. As a result, regions with higher test counts may reflect larger duck farming activity rather than more intensive or targeted surveillance efforts.

Furthermore, the reliance on official monitoring data may introduce biases because certain facilities may not strictly adhere to testing schedules; alternatively, they might delay the submission of test results. Inconsistencies in data collection and reporting could affect statistical analysis reliability. Additionally, although this study incorporated both antigen and antibody testing methodologies, environmental sampling was not performed. The inclusion of environmental sampling—such as detection of viral RNA in nearby water sources or wild bird populations—could provide further insights concerning virus circulation and environmental persistence.

### 4.2 Conclusion

This study assessed the HPAI serological surveillance system across South Korean duck farms, with a focus on the relationships of antigen and antibody test frequencies with epidemic events. The findings indicated that antigen testing serves as a robust predictor of epidemic outbreaks, particularly during high-risk periods, whereas antibody testing provides a Supplementary material tool for tracking long-term exposure. Spatial analysis identified outbreak clusters corresponding to testing intensity, and negative binomial regression confirmed significantly higher test frequencies during high-risk periods. Although test-to-onset intervals have gradually decreased, reflecting improvements in surveillance effectiveness, inconsistencies persist due to factors such as farm depopulation and flock reallocation. Efforts to enhance early outbreak detection and HPAI containment will require refinement of targeted testing protocols and optimization of spatial risk-based surveillance strategies.

Additionally, disparities in testing intensity across regions, particularly in hot-spot areas, highlight the need for further improvements in monitoring strategies. Although antigen testing has been prioritized for detecting active infections, variations in testing frequency across geographical categories suggest that some high-risk areas may experience suboptimal surveillance coverage. Future research should explore the integration of machine learning and real-time monitoring techniques to enhance the predictive capabilities of surveillance systems and optimize testing schedules.

To mitigate the economic and public health impacts of HPAI, future surveillance strategies must incorporate automated detection systems, real-time diagnostic tools, and improved farm-level inspection procedures. By facilitating timely interventions and strengthening South Korea’s preparedness for future outbreaks, the findings of this study support ongoing efforts to enhance avian influenza surveillance systems.

## Data Availability

The original contributions presented in the study are included in the article/Supplementary material, further inquiries can be directed to the corresponding author.

## Supplementary material

Supplementary Data Sheet 1 Summary of HPAI surveillance and outbreak data in South Korean duck farms from 2019 to 2022, including farm locations, testing details (type, timing, and number of tests), and confirmed outbreak records with relevant metadata.

Supplementary Image 1 Supplementary Maps 1–2. Map 1 shows the locations of HPAI outbreaks in duck farms from 2019 to 2022. Map 2 displays the locations of all duck farms included in the study during the same period.
