# Supplementary material for: Evaluation of risk-based antigen and antibody surveillance strategies and their association with HPAI outbreaks in South Korean duck farms
Source: Front Vet Sci. 2025 Jul 3;12:1582269. doi: 10.3389/fvets.2025.1582269 (PMC12268706; doi:10.3389/fvets.2025.1582269)
Supplement: Supplementary Image 1 — Supplementary Maps 1–2. Map 1 shows the locations of HPAI outbreaks in duck farms from 2019 to 2022. Map 2 displays the locations of all duck farms included in the study during the same period. [file Image_1.pdf]

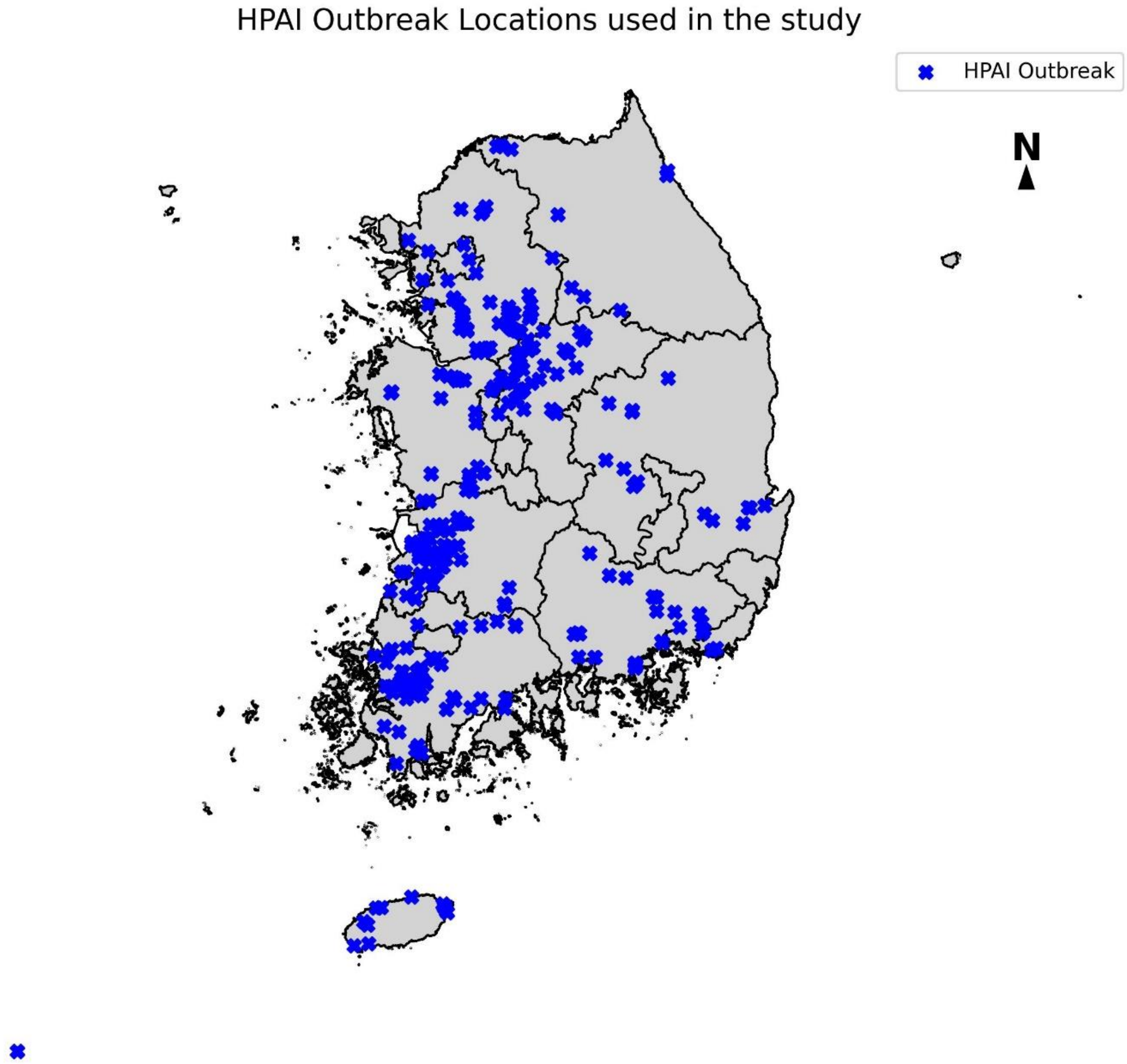

Supplementary Map 1: It shows the HPAI outbreaks in duck farms used in the study from 2019-2022

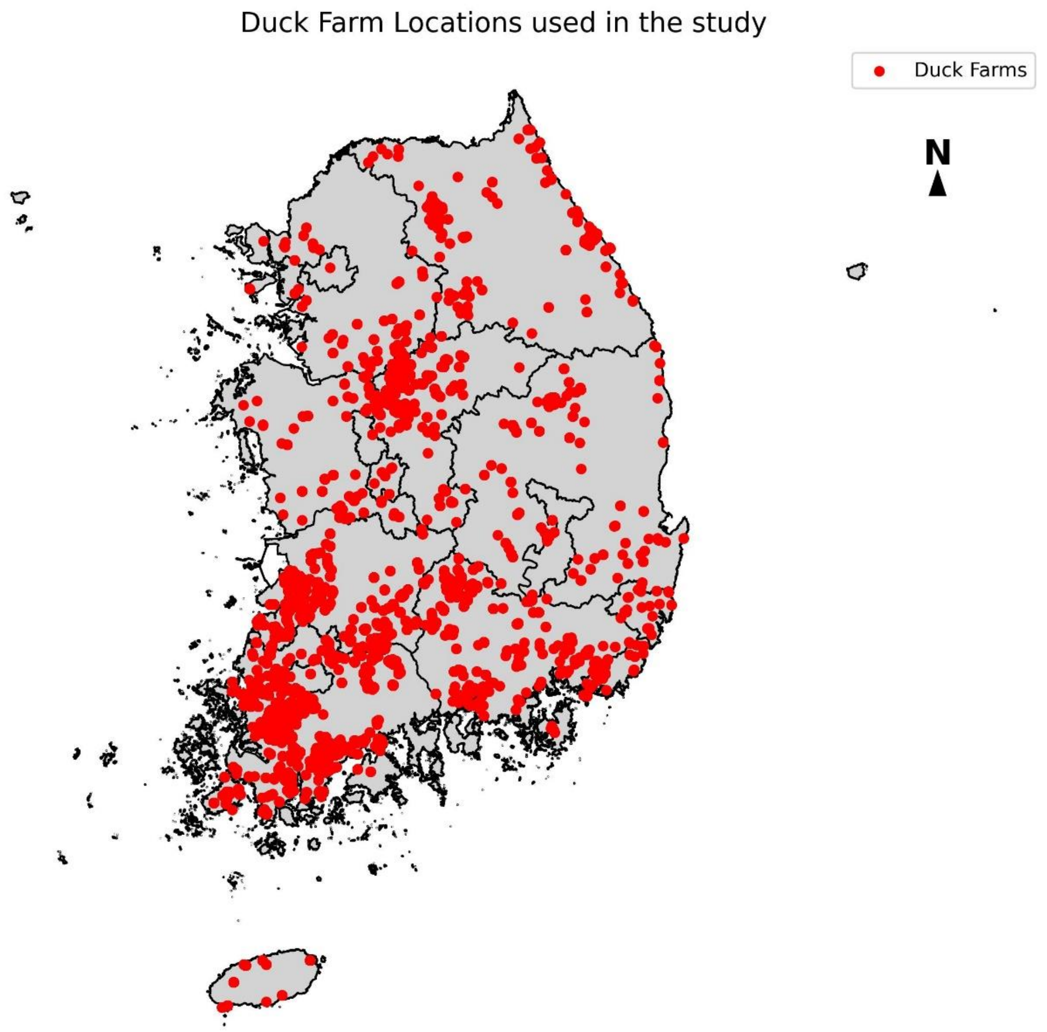

Supplementary Map 2: It shows the duck farms locations used in the study from 2019-2022
